# Obituary for Professor George Vaiopoulos (1943–2023)

**DOI:** 10.31138/mjr.20230925.op

**Published:** 2023-09-29

**Authors:** Meletios Kanakis, Petros P. Sfikakis

**Affiliations:** Department of Paediatric and Adult Congenital Heart Surgery Onassis Cardiac Surgery Centre, Athens, Greece; First Department of Propaedeutic Internal Medicine & Joint Rheumatology Program Medical School, National Kapodistrian University of Athens, Greece

Professor George Vaiopoulos passed away in June 2023. He graduated from the Medical School of Athens and specialised in Internal Medicine at the 1^st^ Department of Internal Medicine at Laikon Hospital. Afterwards, he moved to Montreal in Canada and subspecialised in Rheumatology at the Royal Victoria Hospital (McGill University). He successfully defended his PhD thesis on the biochemistry of haemoglobin O-Thrace. Starting his academic career as a Lecturer, he advanced through the career ladder with distinction, became Professor of Internal Medicine, and, subsequently, the Medical Director of the 1^st^ Department of Internal Medicine at Laikon Hospital, National and Kapodistrian University of Athens Medical School.

This obituary serves as a reminder of his role as a mentor and a teacher for many doctors who served, serve, and will serve in the field of Medicine in Greece.

Professor Vaiopoulos was a gifted hard worker, a person who showed exemplary effort, honesty, and strong moral principles, but above all, a man of integrity. He was always a peacemaker and a source of positivity. He had a remarkable ability to connect with people, and was bestowed with a special gift to be delicate, social, and cheerful with everyone. Professor Vaiopoulos was one of the best clinicians we ever met. He loved helping people, provided treatment, care, comfort, and solace to his patients and their families, asking nothing in return. Throughout his career, he travelled extensively to attend scientific meetings and that gave him a spherical view of Medicine. He made friends and earned the respect of his colleagues nationally and internationally. He was very well known and respected for his clinical studies on Adamantiades-Behçet’s disease.

He was in charge of the training program of medical students and postgraduate medical students and continued working on this project after his retirement, when he became Professor Emeritus. He was one of the founders of the Postgraduate Program for the Experimental Physiology Department of Athens Medical School entitled ‘Molecular and Applied Physiology’.

**Figure:**
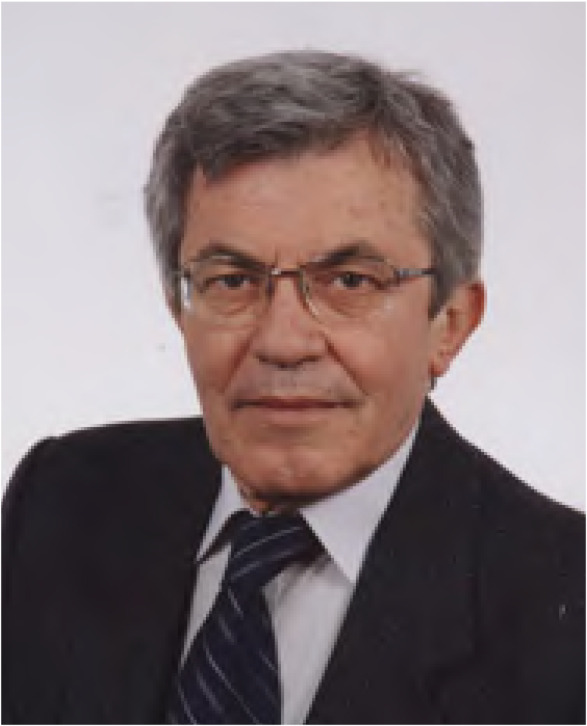


He authored more than 170 international publications, 100 Greek publications, and a significant number of medical books, having given more than 300 lectures in Greek and International conferences.

He leaves behind his wife and son, and two wonderful grandchildren. We hope his family will find the strength to deal with his loss.

We are grateful for having had the opportunity to work with such an outstanding teacher and human being. His death is a great loss to the rheumatological and wider medical community in Greece, but leaves behind a great legacy, since many of his former students now sit in senior positions in Medicine in Greece and are themselves guiding the next generation based on his valuable mentorship.

